# The Lamarckian chicken and the Darwinian egg

**DOI:** 10.1186/s13062-015-0062-9

**Published:** 2015-07-01

**Authors:** Yitzhak Pilpel, Oded Rechavi

**Affiliations:** Department of Molecular genetics, Weizmann Institute of Science, Rehovot, 76100 Israel; Department of Neurobiology, Wise Faculty of Life Sciences & Sagol School, Tel Aviv University, Tel Aviv, 69978 Israel

**Keywords:** Evolution, Darwinian Evolution, Lamarckian Evolution, Modern Synthesis, Weismann Barrier, Epigenetic Inheritance, Transgenerational RNA Interference

## Abstract

**Abstract:**

“Which came first, the Chicken or the Egg?” We suggest this question is not a paradox. The Modern Synthesis envisions speciation through genetic changes in germ cells via random mutations, an “Egg first” scenario, but perhaps epigenetic inheritance mechanisms can transmit adaptive changes initiated in the soma (“Chicken first”).

**Reviewers:**

The article was reviewed by Dr. Eugene Koonin, Dr. Itai Yanai, Dr. Laura Landweber.

## Background

In this commentary paper we wish to use the well-known “Chicken and Egg” paradox as a gateway for discussing different processes of evolution. While in the biological sense this is not a paradox at all, the metaphor is still useful because it allows examining of the distinction between Lamarckian and Darwinian evolution, and specifically, since it enables us to raise an important and unsolved question: “Can the phenotype affect the genotype?” or in other words, “can epigenetics translate into genetics”?

### The only apparent paradox

The “Chicken and the Egg Question” or “Which came first, the chicken or the egg?” is a well-known metaphorical paradox. The original and biologically-relevant question, which has been discussed already by ancient Greek philosophers (*e.g.* “*If there has been a first man he must have been born without father or mother – which is repugnant to nature*” Aristotle), became synonymous with paradoxes in general, since it presents a classical catch: when two events appear to serve both as the cause and the effect of one another it is inconceivable to grasp that any one of them could have preceded the other.

While the metaphysical question might be impossible to resolve, we wish to suggest here that the “Chicken and the Egg” question is not a paradox at all, since precise definition of the question and examination of the possible underling mechanisms of evolution offer a solution. Nevertheless, it is important to discuss why this question is perceived as being paradoxical, and to examine the true mystery that it holds; at the base of this dilemma stands an extremely important and unsolved biological question: “Can the phenotype affect the genotype”? or put differently, “Can epigenetics translate into genetics”?

**Fig. 1:**
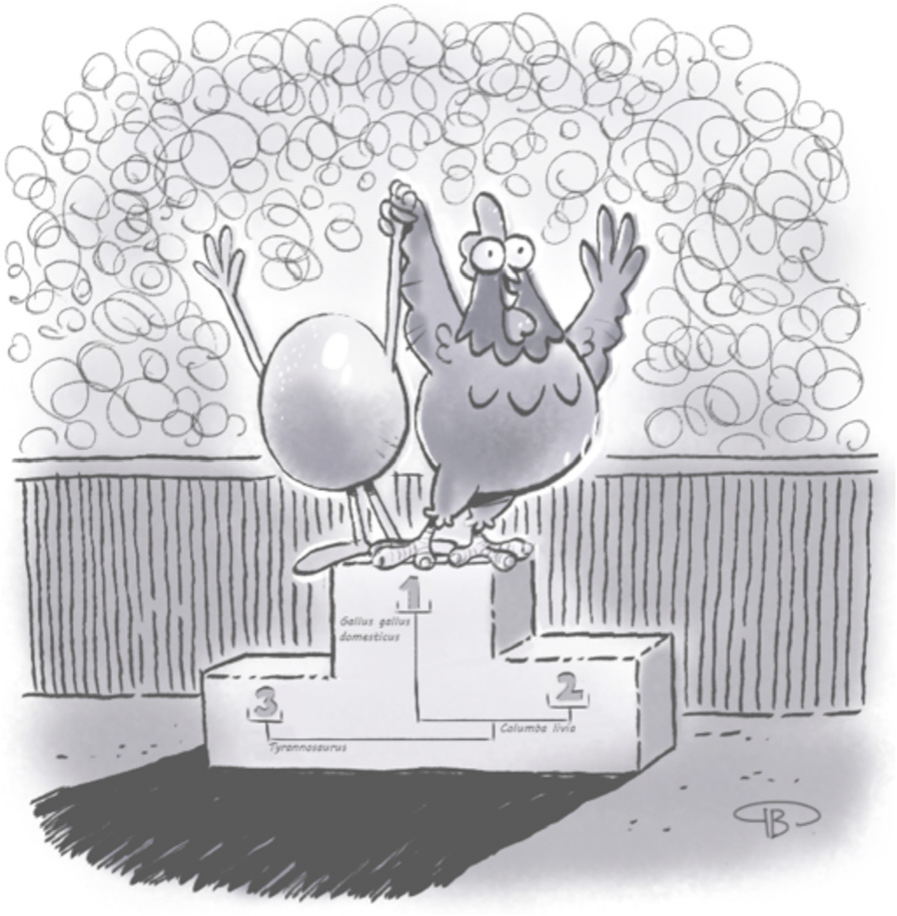
Depending on whether evolution was purely Darwinian or also Lamarckian, both the Egg of the Chicken could “come first”

Asking the question in this manner allows mapping of the “Egg first” vs. the “Chicken first” options onto the distinction between Darwinian and Lamarckian evolution. We wish to suggest that while in many speciation events the “Egg” indeed came first, in some cases, speciation resulted from diverse types of epigenetic inheritance mechanisms, and thus have a “Chicken first” origin (Fig. 1).

While the question is not truly paradoxical, since an imagined “Egg” can precede a “Chicken” or *vice versa* (because organisms are constantly evolving and new species gradually emerge), we ask - which came first? Let us start by defining what we mean when we use the metaphors,”Chicken” and “Egg”.

### The chicken

There is no archetype “Chicken” which is essentially different from its non-chicken ancestor. Richard Dawkins called the human tendency to perceive organisms as members of very discrete “species”, as “the tyranny of the discontinuous mind”. He stressed that all organisms exist in a continuum of evolutionary changes, and wrote “there was never a *Australopithecus* mother who gave birth to a *Homo* child” [4]. Thus, the “new” organism, the “Chicken”, that in our story hatched from a non-Chicken “Egg”, is still, by and large, a “Chicken”, albeit a chicken which harbors certain heritable phenotypical changes that distinguishes it from its ancestor. The formal, dictionary definition of a “Species” – whether or not the progeny can produce fertile offspring with members of its parents’ specie – is irrelevant to this discussion.

### The egg

To deal with the evolutionary mechanism at the heart of this apparent paradox, we wish to only consider here organisms that have somatic cells and dedicated germ cells. The “Egg” in this manuscript, is a germ cell, a sperm or an oocyte. According to the Modern Synthesis only mutations that occur within the germline are inherited to next generations.

### Where did the genetic change originally occur, in the Soma or in the Germline?

Our original “Chicken and Egg” question can now be reframed as: did the heritable change(s) that gave rise to the first Founding Individual in a given speciation event originate in the germline of its parent or in the soma of that individual. Or, more specifically: “Where did the genetic change originate, in the soma of the hen, or of the rooster, in the eggs its mother or in the sperm of its father”?

An “Egg first” solution to the so-called paradox is certainly easy to imagine, compatible with the “Modern Synthesis” between Darwin and Mendel, and probably explains many “speciation” events; in that respect the Egg indeed “came first” in most cases. For the second scenario to be considered, transgenerational inheritance of somatically acquired traits, via epigenetic processes, must take place. Thus the “Chicken first” situation involves evolutionary mechanisms similarly to those envisioned by Lamarck. Evolution according to Lamarck, as described 50 years before the publication of Darwin’s work, is driven by the inheritance of acquired characteristics. According to Lamarck, organisms adapt by developing new variations in response to changing environments, and these new adaptive traits become heritable. Because of the apparent teleological nature of his theory, since it appears to clash with Mendelian genetics, and because no mechanism that enables inheritance of acquired traits was known, Lamarck’s theory was considered, for 200 years, to be completely wrong (E. V [12]).

### “Can epigenetics translate into genetics?

For the sake of accuracy, and although certain historians consider it to be his “greatest mistake”, it is important to remember that at the late stages of his life, Darwin accepted the notion of inheritance of acquired traits [3]. Thus, the old Darwin at least, might have tolerated a “Chicken first” explanation as well. It was August Weissman who hypothesized the existence of a barrier to transfer of genetic information between the soma and the germline. This distinction, in theory at least, made Lamarckism untenable [8, 22]. Also, it must be emphasized that the “Chicken or Egg” question as we define it here (germline Vs. soma), is valid even when all agree that Natural Selection and Drift are the primary processes by which evolution advances. Responses to challenges, and epigenetic changes, which could originate also in somatic cells that interact with the environment, can be selected and maintained similarly to random mutations in the DNA ([2, 8]; E. V. [10, 13]).

While classic DNA-based Mendelian genetics supports the existence of August Weismann’s theoretical barrier between the soma and the germline, and thus “Egg first” solutions only, in recent years discovery and characterization of epigenetic mechanisms that enable transmission of somatically acquired traits across generations may support “Chicken first” scenarios as well. Since this is not an exhaustive review of the subject, we will not get into the details of the different mechanisms, but will simply note that these include epigenetic mechanisms that affect the chromatin, and may or may not be segregated with the chromosomes according to Mendel’s laws (DNA methylation, histone modifications) ([6]; Katz, Edwards, Reinke, & Kelly, [9, 14];; Dias & Ressler 2014), epigenetic mechanisms that “flirt” (interact) with the DNA, such as non coding RNAs-mediated gene regulation ([1, 5, 15, 17, 18]; Rechavi, Minevich, & Hobert, [19, 21]), and DNA-independent inheritance mechanisms (*e.g.* prions, protein feed back loops, hormones, metabolites) [7, 8].

For speciation to occur as a consequence of Chicken-first epigenetic responses, two barriers need to be crossed: the epigenetic to genetic, and the somatic to germline. Crossing the first barrier means that certain inherited epigenetic effects must be replaced by permanent, genome-hardwired changes. Crossing the second barrier amounts to propagation of transgenerational changes that occurred first in the soma into the germline. We can envision two alternative tracks that would lead from epigenetic somatic changes to genetic germline changes.

$$ \begin{array}{c}\hfill \mathrm{Somatic}\kern0.5em \mathrm{epigenetic}\to \mathrm{germ}\hbox{-} \mathrm{line}\kern0.5em \mathrm{epigenetic}\hfill \\ {}\hfill \downarrow \kern0.85em \downarrow \hfill \\ {}\hfill \mathrm{Somatic}\kern0.5em \mathrm{genetic}\kern.75em \to \mathrm{germ}\hbox{-} \mathrm{line}\kern0.5em \mathrm{genetic}\hfill \end{array} $$

The more plausible track appears to be one in which the epigenetic change is first transferred as is from the soma into the germline (*e.g.* in the case of small non-coding RNA in worms), and at a later stage is assimilated and replaced by a genetic change. The alternative is that the somatic epigenetic change could first be assimilated into the soma’s genome and only later be transferred as such into the germline (a scenario with no current supporting data).

Assimilation of epigenetic changes in the genome might be achieved via different mechanisms (E. V [11, 20]). For example, if a gene were silenced by epigenetic means for multiple generations (heritable RNA interference for instance, or repression of transcription by heritable changes to the chromatin or DNA methylation), the gene could become more prone to accumulate mutations in its DNA sequence, some of which could ultimately silence it genetically. Indeed, mounting evidence suggests that such a link exists between transcription and mutation accumulation [24]. While similar mechanisms have not yet been shown to occur in multicellular organisms, in ciliates, non-coding RNAs can directly guide DNA rearrangements and serve as a template for assimilation of mutations in the somatic genome (Nowacki, Shetty, & Landweber, [16]). Until now However, such rearrangements were not found to be transferred to the germline genome.

## Conclusions

There are several apparent “Chicken and Egg Paradoxes” in biology, most notably, the evolution of the translation system. The highly complicated translation system (RNAs and proteins) is needed for its own creation [23]. We suggest that the original “Chicken or Egg” dilemma (how did chicken come to be?) is not a paradox, it is explained by evolution, and that each evolutionary change could map to either a pure Darwinian world (or “Weissmanian” really), in which the metaphorical “Egg” must have preceded the “Chicken", or to a “Lamarckian” world in which the metaphorical chicken “comes first”.

Is there evidence to suggest that inheritance of acquired traits has actually allowed “Chickens” to precede “Eggs”? How prevalent are such instances?

This is an exciting and very active field of research, and a long road is ahead of us before the contribution of epigenetic processes to evolution could be assessed.

## Reviewers’ comments

### Referee #1

Dr. Eugene Koonin. This is, by any account, an insightful essay. The parallelism between “chicken-or-egg”-type paradoxes and the Lamarckian vs Darwinian (historically more precise could be Weissmanian, as the authors rightly note) is obvious once stated but I think has not been explicitly discussed before. The discussion in the paper centers on the interaction between germ line and soma but towards the end the authors notice that the complementarity between the two modalities of evolution is likely to be a general phenomenon relevant to all kinds of organisms. This rings true to me. I still wonder, though, how important are epigenetic phenomena in unicellular organisms. They definitely possess Lamarckian-type evolutionary mechanisms (CRISPR-Cas is the prime example) but do they have epigenetics sensu stricto? Perhaps, the authors could comment on that. A few highly relevant references seem to be missing: Koonin EV. Does the central dogma still stand? Biol Direct. 2012 Aug 23;7:27 (sorry to honk my own horn here but this paper does discuss explicit mechanisms whereby phenotypic changes could be assimilated in genomic mutations) Shapiro JA. How life changes itself: the Read-Write (RW) genome. Phys Life Rev. 2013 Sep;10(3):287–323.

### Response to referee #1

We thank Dr. Koonin for his helpful and generous comments. We did not elaborate in the paper regarding the relevancy of epigenetic mechanisms to evolution in unicellular organisms. However, we believe epigenetic inheritance could play a role in shaping the evolution of unicellular organisms as well. Indeed, while the CRISPR-cas system in bacteria (as mentioned by Dr. Koonin) is “Lamarckian”, it is not an “epigenetic” system par excellence. Nevertheless, other forms of epigenetic inheritance exist in single cell organisms, for example inheritance of prions in yeast, epigenetic inheritance of chromatin modifications that has been specifically studied in fission yeast, and inheritance of regulatory RNAs in ciliates. Whether this form of inheritance is important for their evolution is still an open question. We gladly added the additional references that Dr. Koonin rightly suggested.

### Referee #2

Dr. Itai Yanai. In this manuscript, Pilpel and Rechavi make the clever connection between Darwinian and Lamarckian mechanisms and the famous Chicken and Egg paradox. This allows them to nicely frame the question of where does novelty ultimately arise: in the germ line or in the soma? Since the Modern Synthesis a century ago, it has been held that novelty arises as mutations in the germ line, however exciting research over the past few years has provided evidence that changes occurring in the soma can also be heritable across generations. I commented on an earlier version of this manuscript and am even more pleased with its current state. One limitation of the analogy that may be worth pointing out again is that under the chicken-first scenario, really only one cell – and not the entire organism - has the ‘chicken-phenotype’.

One issue that I feel is missing in the discussion is the role that any somatic epigenetic change plays in adaptation. In other words, are the changes necessarily of any consequence? If, through interaction with the environment, an adaptive somatic change may occur and become heritable, the details of such a mechanism would be extremely interesting. In a related note, the CRISPR system in bacteria can be said to be Lamarckian since the adaptive changes to the genome are inspire by the environment (the attacking viruses): did proto-chickens have a similar mechanism?

Finally, in the two paths imagined by the authors, why does an epigenetic mutation precede a somatic mutation? It seems plausible that there is a third path that simply starts with a somatic genetic change and leads to a germline genetic change.

### Response to referee #2

We thank Dr. Yanai for his nice comments and suggestions. We think that an entire organism could have ‘chicken-phenotype’ due to a synchronized epigenetic response, which does not have to be limited to one particular cell. Synchronization of epigenetic responses could occur, in particular, if the epigenetic change takes place while the organism develops. For example, if a gene is epigenetically modified (silenced, for example) early in development, and the silenced state would be maintained after cell division. Dr. Yanai asks in addition whether heritable epigenetic changes are necessarily of any consequence. We think that heritable epigenetic changes will be selected, much similarly to bone fide genetic changes, and thus non-adaptive changes, if significant enough, would be eliminated. Additionally, epigenetic changes, just as genetic mutations, that are neutral or that bear little phenotypic effect could be carried over by drift.

### Referee #3

Dr. Laura Landweber. This brief essay blends philosophy and history of science with recent review. One hypothesis is that some cases of speciation may result from epigenetic mutations, or alterations in the interpretation of the genome. This is, of course, consistent with a large body of literature documenting the importance of regulatory changes in evolution, but merging that field specifically withepigenetics seems a new proposal. Pilpel and Rechavi don’t have specific examples to offer yet, but propose this basic hypothesis. Epigenetics can certainly contribute quite a bit to variation, within and between species, but a few evolutionary biologists are just beginning to think about the layer of variation that epigenetics introduces. As to ciliate systems, an expertise of my lab, we have never observed mutations in the germline that correspond to mutations that first arise by manipulation of the somatic epigenome. Thus, like regulatory changes, the epigenetic changes are still at the level of the interpretation of the germline information in the soma.

### Response to referee #3

We thank Dr. Landweber for reviewing the paper, and specifically for providing insights regarding interactions between epigenetic inheritance and germline mutations in cilliates. Indeed it would be very exciting if evidence were found to support the theory that somatic epigenetic mutations can precede (and direct) germline mutations.
